# Point-of-Care Ultrasound Findings in Occlusive Iliac Vein Thrombus During Pregnancy: A Case Report

**DOI:** 10.5811/cpcem.6658

**Published:** 2024-06-14

**Authors:** Donald Pettet, John Forrester, Mathew Nelson, Tanya Bajaj

**Affiliations:** North Shore University Hospital, Department of Emergency Medicine, Manhasset, New York

**Keywords:** *deep vein thrombosis*, *DVT*, *pregnancy*, *point-of-care ultrasound*, *case report*

## Abstract

**Introduction:**

Diagnosing deep venous thromboses and venous thromboemboli (DVT/VTE) in pregnant patients presents a unique challenge for emergency physicians. The risk of DVT/VTE increases during pregnancy, and the potential consequences of misdiagnoses are severe. Point-of-care ultrasonography (POCUS) is frequently a first-line diagnostic imaging modality. However, recent studies have shown a high incidence of thromboses proximal to the common femoral vein during pregnancy, and these would not be visualized using compressive ultrasonography, which traditionally can only visualize thromboses distal to the femoral vein.

**Case Report:**

A 38-year-old female, 25-weeks primiparous, presented to the emergency department with a three-day history of left lower extremity swelling. Point-of-care three-point compression testing was used to evaluate for a DVT; however, no thrombus was visualized. Given high clinical suspicion, color and spectral Doppler testing were performed and demonstrated turbulent flow and reduced respiratory variation in the common femoral vein. This prompted further additional testing for a proximal DVT using magnetic resonance venography, which revealed an occlusive left external iliac thrombus. The patient was subsequently started on daily subcutaneous enoxaparin and discharged home with close follow-up.

**Conclusion:**

Emergency physicians play a critical role in evaluations for the presence of DVT/VTE, particularly in pregnant patients. We endorse the use of POCUS with three-point compression testing, as well as color and spectral Doppler imaging, to help identify proximal DVTs in this patient population. This case report can aid physicians in the diagnosis of this pathological condition that if left untreated can have severe consequences.

CPC-EM Capsule What do we already know about this clinical entity?

*Pregnant patients are at increased risk for deep vein thromboses (DVT) proximal to the femoral vein.*

What makes this presentation of disease reportable?

*While three-point compression testing is frequently applied in the emergency department, this case highlights false-negative results due to a proximal DVT.*

What is the major learning point?

*Three-point compression testing may lead to false-negative results in pregnant patients due to increased rates of thromboses proximal to the femoral vein.*

How might this improve emergency medicine practice?

*Consideration for proximal DVT in pregnant patients may help avoid adverse outcomes related to false-negative, three-point compression testing.*

## INTRODUCTION

Diagnosing deep venous thromboses (DVT) and venous thromboemboli (VTE) in pregnant patients presents a unique challenge for emergency physicians. Pregnancy increases the risk of DVT/VTE. The potential consequences of misdiagnoses are severe and include DVT/VTE recurrence, post-thrombotic syndrome, pulmonary embolism, and even death.^1^ Venous ultrasonography is a first-line diagnostic imaging modality in the emergency department (ED) for patients with a moderate to high pretest probability for DVT/VTE.^2^ However, recent studies have shown a higher incidence of thromboses proximal to the common femoral vein during pregnancy.^3^ For this reason, in cases where clinical suspicion remains high despite negative three-point compression testing, adjunctive ultrasound imaging modalities and more comprehensive imaging may be required.

In this report, we present a case of a previously healthy pregnant woman in her second trimester with a DVT within the left external iliac vein, which was not initially detected with point-of-care ultrasonography (POCUS) and three-point compression testing. With limitations regarding the use of ultrasonography to detect thromboses proximal to the common femoral vein, this case underscores the importance of considering this entity in pregnant patients with suspected DVT/VTE.^4^

## CASE REPORT

A 38-year-old female presented to the ED with a three-day history of progressively worsening swelling in her left lower extremity. At the time of presentation, she was 25 weeks pregnant and had no significant medical, surgical, or family history. Physical examination revealed circumferential swelling in her left lower extremity extending from the knee to the distal forefoot. Additionally, she exhibited left calf tenderness upon palpation.

To evaluate for the presence of a DVT in her left lower extremity, we used POCUS with three-point compression testing. The femoral and popliteal regions demonstrated normal manual compressibility, and no thrombus was visualized. Despite these findings, the patient’s exam and clinical history raised suspicion for thrombosis. As a result, color and spectral Doppler testing were performed, which revealed turbulent flow and reduced respiratory variation in the common femoral vein. These findings prompted additional testing for a proximal DVT using magnetic resonance venography (MRV), which ultimately revealed an occlusive DVT in the left external iliac vein. The patient was subsequently evaluated by hematology, started on daily subcutaneous enoxaparin sodium injections, and discharged home with close follow-up.

## DISCUSSION

A DVT is considered “proximal” when it is identified within the popliteal, femoral, or iliac veins of the lower extremity on radiographic imaging. The initial presentation for patients with proximal DVTs can vary significantly, but common symptoms include swelling, discoloration, and cramping sensations within the calf and thigh of the effected limb. In the ED, the initial diagnostic approach involves an assessment of pretest probability with consideration for the patient’s history, genetic and acquired risk factors, and signs and symptoms. One risk factor of particular importance to the emergency physician is the association of DVT/VTE with pregnancy.

Observational studies and meta-analyses have established pregnancy as a risk factor for DVT/VTE, with as many as 1.0–1.8% of pregnant patients developing this condition.^4^ This thromboembolic risk increases throughout pregnancy to nine-fold during the third trimester, and peaks two to six weeks postpartum when the risk is 80 times higher than in non-pregnant patients.^5^ It is important to note that unlike in men and non-pregnant women, pregnant patients are more likely to develop proximal rather than distal thromboses. Previous studies investigating the anatomic distribution of DVTs in pregnant patients found thromboses to more commonly be left-sided (84%) and restricted to the iliofemoral (64%) and iliac (12%) veins.^6^ Given that 9.3% of pregnancy-related deaths are due to pulmonary emboli, the importance of identifying such thromboses in this patient population cannot be overstated.^7^

In pregnant patients, the formation of DVT/VTE is heavily influenced by physiologic hypercoagulability and intra-abdominal anatomical changes. Pregnancy is characterized by a hypercoagulable state caused by an increase in clotting factors, a decrease in natural anticoagulants, and alterations in fibrinolytic activity.^8^ These changes are thought to be protective against hemorrhage during delivery, but they also predispose patients to thromboses. The approximation of the gravid uterus with the vertebral bodies due to increased lordosis also predisposes pregnant patients to thromboses through venous compression.^9^ The influence of these anatomical changes is highlighted in cases of aortocaval compression when pregnant patients are lying supine.

Other anatomical influences may also play a role. For example, May-Thurner syndrome is a condition in which compression of the left common iliac vein by the overlying right common iliac artery may lead to inflammation, sclerosis, venous stasis, and thromboses. May-Thurner syndrome has been identified in over 30% of all-comers previously diagnosed with left lower extremity DVT and is often overlooked.^10^ Other risk factors for DVT/VTE in pregnancy include immobility, advanced maternal age, obesity, smoking, multiple gestations, and a personal or family history of thromboembolic disease. It is important for emergency physicians to be aware of these risk factors and maintain a high level of suspicion for DVT/VTE in pregnant patients presenting with lower extremity swelling or pain.

The initial imaging test of choice when evaluating a pregnant patient for a DVT is venous ultrasonography. Traditionally, the initial diagnostic imaging modality of choice has been the complete duplex ultrasound. However, with the emergence of POCUS, three-point compression testing has been employed in the ED to evaluate patients for DVTs, with advocates highlighting improved times to study completion, decreased costs, and the avoidance of unnecessary transfers and treatments.^11^ ^–^ ^13^ This method involves evaluations of the common femoral vein one to two centimeters above the bifurcation with the saphenous vein, the common femoral vein at the bifurcation with the saphenous vein, the femoral vein at the bifurcation with the deep femoral vein, and the popliteal vein.

Loss of compressibility is considered the most reliable indicator of a DVT, and color Doppler imaging may be used to differentiate between complete and incomplete occlusions. Multiple studies have validated this approach and demonstrated false negative results in as little as 1.4%.^14^ ^,^ ^15^ A recent systematic review and meta-analysis by Hercz et al additionally found a pooled sensitivity of 90% and specificity of 95% using emergency physician-performed ultrasound evaluations for DVTs.^16^ Unfortunately, studies evaluating the use of three-point compression testing during pregnancy are not available. Importantly, the American College of Obstetrics and Gynecology guidelines recommend close follow-up with repeat imaging at three and seven days if ultrasound evaluations are negative and an iliocaval thrombosis is not suspected.^7^ This case highlights the diagnostic challenge that emergency physicians may encounter, as three-point compression testing can yield negative results even in the presence of an occlusive iliocaval thrombus.

Given the possibility of false-negative results with three-point compression testing in pregnant patients, POCUS evaluations should incorporate color and spectral Doppler studies of the common femoral veins. Color Doppler enables clinicians to visualize turbulent flow patterns, also known as “rouleaux,” while spectral Doppler can identify changes in respiratory phasicity, suggesting a venous occlusion proximal to the area of insonation.^17^ Under normal conditions, spectral Doppler testing should demonstrate phasic variations in waveform throughout the cardiac cycle due to respiration. An abnormal spectral Doppler waveform that is flat and continuous indicates decreased respiratory variation and should raise concern for a venous obstruction proximally.^18^ Importantly, to obtain accurate results physicians should ensure that the angle of incidence is below 60 degrees and compare results to the contralateral extremity.

**Image 1.**
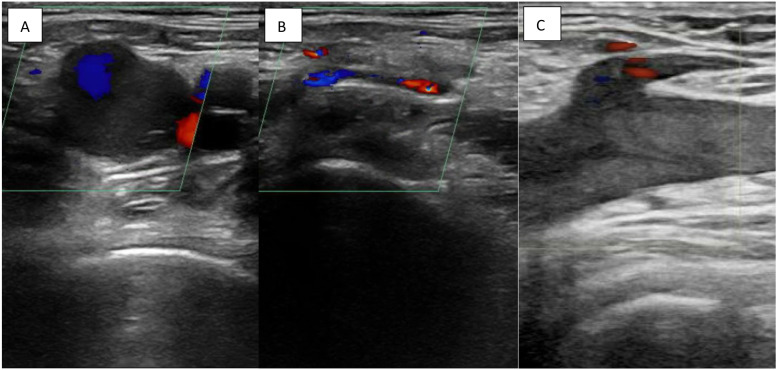
Point-of-care ultrasound images of the left common femoral vein in transverse view without (A) and with (B) compression, and in long view (C). Notably, images A and C demonstrate sluggish venous flow with a lack of directionality in the common femoral vein using color Doppler.

**Image 2.**
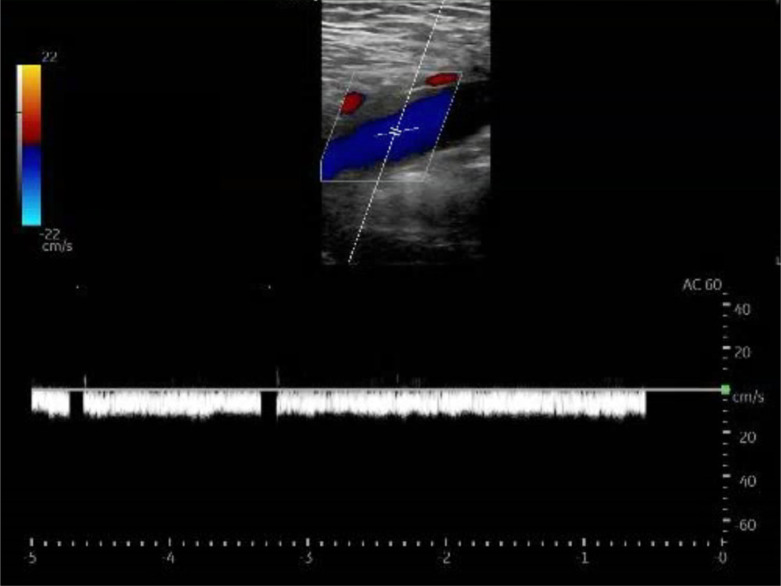
Point-of-care ultrasound image of the left common femoral vein in longitudinal view demonstrating lack of normal respiratory phasicity with spectral Doppler.

**Image 3.**
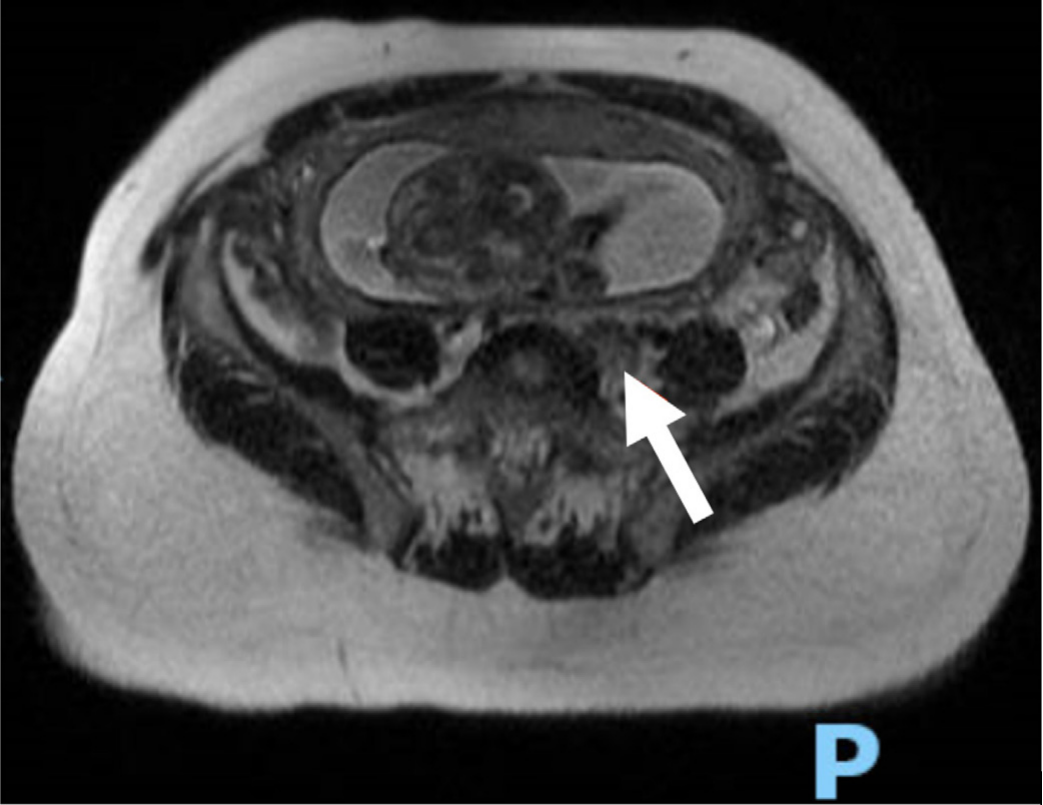
Magnetic resonance venogram image of the left external iliac vein demonstrating a deep vein thrombosis (white arrow).

Despite negative results with three-point compression testing, circumferential leg swelling in this patient’s case prompted color and spectral Doppler assessments for a proximal, iliocaval thrombus. These studies ultimately demonstrated turbulent flow patterns and decreased respiratory phasicity (Images 1 and 2). If there is evidence of a proximal venous obstruction on ultrasound imaging, computed tomography and MRV have been established as the imaging modalities of choice due to limitations of duplex ultrasonography above the inguinal plane.^19^ ^,^ ^20^ In this patient’s case, magnetic resonance imaging was obtained and identified a DVT within the left external iliac vein (Image 3). Overall, this case highlights the importance of investigations for thromboses proximal to the common femoral vein in pregnant patients when suspicion is high, even when three-point compression testing is negative.

## CONCLUSION

Emergency physicians play a critical role in the evaluation of high-risk patients for the presence of DVT/VTE, particularly in pregnant patients who may have familial, genetic, or anatomic factors that increase their risk for thromboses. Clinicians evaluating pregnant patients should maintain a high degree of clinical suspicion and have a low threshold to obtain comprehensive ultrasound studies and magnetic resonance venography when necessary. The information provided in this case underscores the importance of considering DVTs proximal to the common femoral vein in pregnant patients and may aid clinicians in the diagnosis of this pathological condition, which if left untreated can result in serious consequences, including DVT/VTE recurrence, post-thrombotic syndrome, pulmonary embolism, and even death.
